# Physiologically based pharmacokinetic modeling of intravenously administered nanoformulated substances

**DOI:** 10.1007/s13346-022-01159-w

**Published:** 2022-05-12

**Authors:** Jordi Minnema, Sven Even F. Borgos, Neill Liptrott, Rob Vandebriel, Christiaan Delmaar

**Affiliations:** 1 National Institute for Public Health and the Environment, Bilthoven, The Netherlands; 2 SINTEF, Trondheim, Norway; 3 Immunocompatibility Group, Department of Pharmacology and Therapeutics, Institute of Systems, Molecular and Integrative Biology, University of Liverpool, Liverpool, UK

**Keywords:** Physiologically based pharmacokinetic modeling, Nanobiomaterials (NBMs), Biodistribution, Bayesian parameter estimation

## Abstract

The use of nanobiomaterials (NBMs) is becoming increasingly popular in the field of medicine. To improve the understanding on the biodistribution of NBMs, the present study aimed to implement and parametrize a physiologically based pharmacokinetic (PBPK) model. This model was used to describe the biodistribution of two NBMs after intravenous administration in rats, namely, poly(alkyl cyanoacrylate) (PACA) loaded with cabazitaxel (PACA-Cbz), and LipImage™ 815. A Bayesian parameter estimation approach was applied to parametrize the PBPK model using the biodistribution data. Parametrization was performed for two distinct dose groups of PACA-Cbz. Furthermore, parametrizations were performed three distinct dose groups of LipImage™ 815, resulting in a total of five different parametrizations. The results of this study indicate that the PBPK model can be adequately parametrized using biodistribution data. The PBPK parameters estimated for PACA-Cbz, specifically the vascular permeability, the partition coefficient, and the renal clearance rate, substantially differed from those of LipImage™ 815. This emphasizes the presence of kinetic differences between the different formulations and substances and the need of tailoring the parametrization of PBPK models to the NBMs of interest. The kinetic parameters estimated in this study may help to establish a foundation for a more comprehensive database on NBM-specific kinetic information, which is a first, necessary step towards predictive biodistribution modeling. This effort should be supported by the development of robust in vitro methods to quantify kinetic parameters.

**Graphical abstract:**

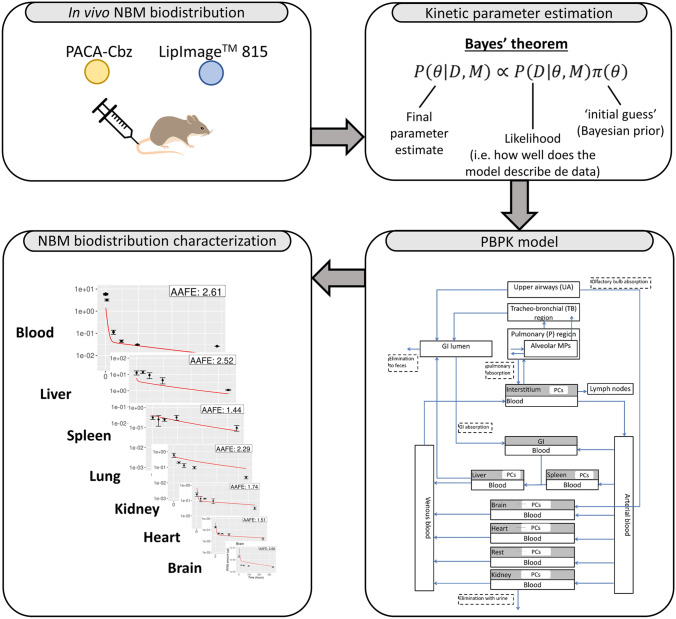

**Supplementary information:**

The online version contains supplementary material available at 10.1007/s13346-022-01159-w.

## Introduction

Nanotechnology is rapidly gaining popularity in the field of medicine [1]. In medical settings, nanotechnology typically refers to the application of nanobiomaterials (NBMs) for surgical and medical treatments of patients. NBMs offer the opportunity of altering the associated pharmacokinetic profile of a drug, by improving dissolution in biological matrices, and prolonging systemic circulation time [2]. Since NBMs can be designed with a variety of physical–chemical characteristics, they can also be used in a variety of applications [3]. One way of applying NBMs is to encapsulate a drug with a NBM. Such encapsulated formulations can delay the elimination of the drug from the body, change their solubility, or lower their toxicity [4]. Additionally, NBMs are particularly useful for targeted drug delivery, since the modified physical–chemical properties make it possible to target specific tissues or organs [5]. In fact, the application of NBMs is not limited to drug delivery, as they have also been used for medical imaging and diagnosis [6].

For many applications, NBMs are designed to alter the biodistribution of a substance of interest. In order to investigate whether designed NBMs exhibit the desired distribution characteristics, in vitro tests [7, 8] may be useful. For example, an in vitro assay has been developed to investigate the relation between phagocytic uptake and blood clearance [9]. However, the measured in vitro kinetics do not always translate to in vivo distributions. Moreover, they are usually specific to certain NBMs, and cannot always be used to infer generalities on the biodistribution of other NBMs. Hence, quantitative in vitro-to-in vivo extrapolation (QIVIVE) remains challenging. As a consequence, researchers often still rely on animal experiments in order to investigate the biodistribution of NBMs. For instance, the biodistributions of various NBMs involving chemotherapeutic drugs have been investigated in mice, such as plitidepsin [10], cisplatin [11], and irinotecan [12]. Furthermore, the biodistribution of nanoformulated radiolabels was studied in mice [13] and rats [14].

A particularly useful tool to quantitatively describe the biodistribution of NBMs is that of physiologically based pharmacokinetic (PBPK) modeling. PBPK models represent particular body tissues and organs with interconnected compartments that exchange material with rates determined by physiological transport processes. PBPK models may be used to infer delivered doses at a tissue level, extrapolate experimentally determined biodistribution to different exposure conditions, or even to predict the distribution of NBMs based on limited a priori kinetic information. A crucial step in effectively using PBPK models is finding appropriate values for the parameters defining the PBPK model (i.e., its “parametrization”). In practice, the parametrization is determined from experimental in vivo biodistribution data using a model-fitting procedure. The specific numerical values of the parameters estimated with such a procedure may be subject to large uncertainties, due to variability and imprecision in the data and due to a lack of identifiability of parameters. Also, in vivo biodistribution studies are expensive in terms of resources as well as animal life. Potentially, parametrization of PBPK models could be greatly aided with adequate in vitro testing systems that are predictive of specific kinetic processes or even PBPK model parameters [15]. Firstly, in vitro measurements can serve as initial (a priori) information in the interpretation of in vivo data by setting ranges on possible parameter values. Such information will significantly improve the parameter estimation from limited data. Second, and more importantly, in vitro kinetic information for critical, NBM-dependent model parameters can aid in the extrapolation of a PBPK model from one (well parametrized) NBM to another (i.e., read-across). In this way, in vitro testing could assist in the effort to replace, reduce, and refine (i.e., 3Rs principle) animal studies that are still widely used as a first step towards clinical trials involving NBMs.

To effectively implement a pipeline that includes in vitro determination of kinetic parameters, several aspects need to be established. First, relevant in vitro systems must be available to represent physiological aspects that can be adequately quantified and included in a PBPK model. Second, the modeling should be able to effectively incorporate a priori information obtained from the in vitro system. Third, with respect to the objective of read-across of PBPK modeling, cataloging of critical kinetic model parameters is required. This third step includes the identification of generic parameters that do not vary much between NBMs, parameters that are specific to an NBM and may vary strongly between NBMs, and parameters for which the PBPK model is not very sensitive, so that acquiring very precise information for these parameters is not useful. Such a catalog of PBPK parameter information requires an extensive set of studies on a wide variety of NBMs. To date, few studies have been published in which PBPK models have been parametrized to describe the biodistribution of NBMs. For example, Dogra et al. [16] implemented a PBPK model to describe the biodistribution of nanoparticles based on their diameter, whereas Fallon et al. [17] used a PBPK model to describe the concentration profiles of amitriptyline nanoparticles (antidepressant drug). Furthermore, Rajoli et al. [18, 19] implemented a PBPK model to describe the biodistribution of antiretrovirals with physicochemical properties that are compatible with nanoformulations. PBPK models have been applied relatively often to describe the biodistribution of liposomal NBMs such as Doxil® [20, 21] and AmBisome® [20, 22]. However, this dataset is still too limited to serve as a basis for a robust parameter cataloging. Additional PBPK studies are needed.

The present study therefore implements a PBPK model and parametrizes the model based on in vivo biodistribution data for two different NBMs. The first NBMs consists of poly(alkyl cyanoacrylate) (PACA) nanoparticles (NPs) loaded with the chemotherapeutical drug, cabazitaxel. The second NBMs consists of nanostructured lipid carriers loaded with the fluorescent dye IR780. In order to estimate the PBPK model parameters, in vivo biodistribution data of the two substances in rat were used. The biodistribution of the loaded substances is hypothesized to follow that of the encapsulating NBM. Model parameters characterizing the distribution of these NBMs are estimated and analyzed with respect to the extent with which they may be properly estimated from the data and the sensitivity of the model to them. Model parameters are compared cross-NBM to build hypotheses on generic model parameters that may be representative of larger groups of NBMs. To the best of our knowledge, this is the first study to parametrize PBPK models to simulate the biodistribution of PACA loaded with cabazitaxel and LipImage™ 815.

### Materials and methods

#### In vivo biodistribution datasets

In order to parametrize a PBPK model to describe the biodistribution of nanoparticles, dedicated in vivo biodistribution data are needed. In this study, two different biodistribution datasets were used, which were both based on studies in rat. The first biodistribution dataset was obtained following intravenous (iv) administration of PACA NBMs loaded with cabazitaxel (PACA-Cbz) [23]. These PACA NBMs can be used as drug-loading carriers since they facilitate targeted delivery of the chemotherapeutic drug to the tumor site [24]. The dataset was obtained by measuring cabazitaxel concentrations in various organs after administration of the PACA-Cbz. The second dataset was obtained following iv administration of LipImage™ 815 [23]. LipImage™ 815 is a novel contrast agent used in near-infrared fluorescence imaging [25]. It consists of nanostructured lipid carriers that are loaded with the fluorescent dye IR780. The concentration of this fluorescent ICG dye was measured in rat organs. Details of both datasets are given below.

##### PACA-Cbz

A biodistribution study has been performed which involved a single administration of PACA NBMs loaded with the drug cabazitaxel (PACA-Cbz) produced by SINTEF (Trondheim, Norway). Cabazitaxel is a chemotherapeutic agent that has been approved by the US food and drug administration (FDA) for the treatment of patients with metastatic prostate cancer. The PACA nanocarriers consisted of poly(2-ethylbutyl cyanoacrylate) (PEBCA). The particle hydrodynamic diameter and zeta potential of the PACA-Cbz NBM were determined using dynamic light scattering (DLS), which resulted in 121.8 nm (z-avg) and − 5.5 mV, respectively [23]. In addition, a polydispersity index (PDI) of 0.14 was measured [23].

In this biodistribution study, PACA-Cbz was administered iv with two distinct cabazitaxel doses of 0.5 μg/g body weight, and 3.5 μg/g body weight. Cabazitaxel concentrations in the blood were measured using a puncture after 1 min, 3 min, 7 min, 15 min, 30 min, 1 h, 4 h, 1 day, 2 days, 4 days, and 14 days. The biodistribution of the encapsulating PACA NBMs was assumed to follow that of the cabazitaxel. Rats were sacrificed at five different time points: 1 h, 1 day, 2 days, 4 days, and 14 days post-exposure. At each time point, four rats were sacrificed. After sacrifice, cabazitaxel concentrations in the blood, liver, spleen, lungs, kidneys, heart, and brain were established. These data were used to parametrize the PBPK models in the present study. Further details on the PACA-Cbz biodistribution will be published by Åslund et al. [23].

##### LipImage™ 815

The biodistribution of LipImage™ 815 has been assessed in a dedicated experiment, which was based on a single iv administration. LipImage™ 815 is produced by the French Alternative Energies and Atomic Energy Commission (CEA) and consists of a nanostructured lipid carrier that is loaded with IR780 [25]. The particle hydrodynamic diameter of LipImage™ 815 was determined using dynamic light scattering (DLS), which resulted in 52.2 nm (z-avg) [23]. In addition, a polydispersity index (PDI) of 0.102 was measured [23]. The zeta potential of LipImage™ 815 is neutral (0 mV) [23].

In the dedicated biodistribution study, three different dose group were used, which corresponded with IR780 doses of 0.046 μg/g body weight, 0.15 μg/g body weight, and 0.46 μg/g body weight. Similar to the PACA-Cbz dataset, IR780 levels were measured in the blood using a puncture 15 min, 30 min, 1 h, 4 h, and 24 h post-exposure. The biodistribution of the lipid NBMs was assumed to follow that of the IR780. Rats were sacrificed at 1 h, 1 day, 2 days, 4 days, and 14 days post-exposure. After the sacrifice, the blood, liver, spleen, lungs, kidneys, heart, and brain were collected for IR780 analysis. Although IR780 levels were also measured in the thymus and the testes, these data were not considered in this study since those organs are not explicitly included in our PBPK model. Further details on this LipImage™ 815 biodistribution dataset will be published by Åslund et al. [23].

Table 1 summarizes the doses and measurement time points of the five biodistribution subsets.

**Table 1:** Summary of the biodistribution datasets used to parametrize the PBPK models in the present study

Dataset	Loaded substance concentration (μg/g bw)	Time points of sacrifice (h)
PACA-Cbz	0.5	1 h, 1d, 2d, 4d, and 14d
PACA-Cbz	3.5	1 h, 1d, 2d, 4d, and 14d
*LipImage™ 815*	0.046	1 h, 1d, 2d, 4d, and 14d
*LipImage™ 815*	0.15	1 h, 1d, 2d, 4d, and 14d
*LipImage™ 815*	0.46	1 h, 1d, 2d, 4d, and 14d

#### PBPK model framework

**Fig. 1:**
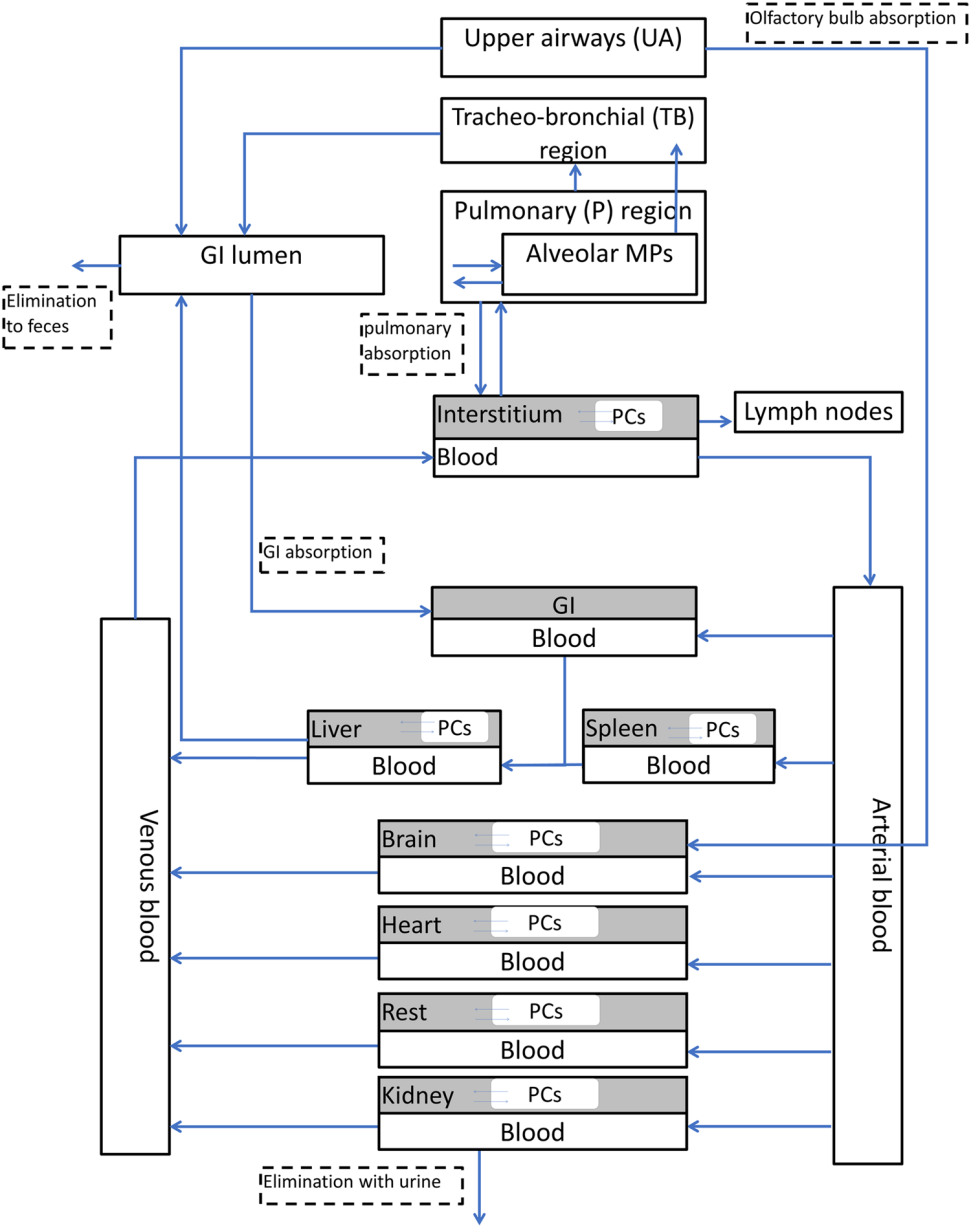
Schematic overview of the PBPK model used in the present study. This PBPK model was adapted from Li et al. [26]

The PBPK model implemented in this study was originally developed by Li et al. [26] to describe distribution of nano CeO_2_ in rat. Fourteen main compartments are included in this model, namely, arterial blood, venous blood, upper airways, tracheobronchial region, pulmonary region, liver, spleen, kidney, heart, brain, gastro-intestinal (GI) tract, and the remaining organs (rest). All compartments are interconnected via the blood circulation. In addition, eight organ compartments comprised a sub-compartment that corresponds with the phagocytizing cells. Finally, four different clearance routes are included in this PBPK model: the lymphatic system, the urine, the feces, and liver metabolism. A schematic overview of the PBPK model is shown in Fig. 1. Since the PBPK model was applied to simulate biodistributions after iv administration, the total injected dose was modeled to be deposited in the arterial blood compartment.

#### PBPK model parameter estimation

To estimate the PBPK model parameters, a Bayesian method was adopted. Bayesian parameter estimation has the advantage that prior information on possible parameter values can be taken into account from previous experiments or literature. Furthermore, new information from various sources (e.g., expert judgement, in vitro test results, kinetic parameters) can be incorporated via the prior when this information becomes available. This essentially allows one to refine and update model parameter information when new experimental datasets are acquired. Bayesian parameter estimation approaches are based on Bayes’ theorem, which is formulated as follows:

1 $$P\left(\theta\vert D\right)=\frac{P(\theta)\cdot P(D\vert\theta)}{\int P(\theta')\cdot(D\vert\theta')d\theta'}$$

$\theta$ represents the set of model parameters and $D$ represents an observed data (e.g., measured concentrations or amounts in different organs). On the left-hand side of the equation is the posterior likelihood distribution, $P\left(\theta |D\right)$, the likelihood of parameter values $\theta$ given the data $D$. This can be interpreted as a probability distribution of the values for the parameter $\theta$, i.e., as the parameter estimate. $P(\theta )$ is the prior distribution, which expresses knowledge on the parameter values $\theta$ before evidence $D$ is considered (e.g., evidence available from previous studies, based on read-across from similar materials or obtained from a representative in vitro test system). $P(D|\theta )$ is the data likelihood. This expresses how well the PBPK model describes the observed data $D$ for a particular set of parameters $\theta$. $\int P(\theta')\cdot P\left(D\vert\theta'\right)d\theta'$ represents the total likelihood of the data $D$. It is used as a normalization factor for the posterior.

Generally, solving Eq. (1) is complicated in view of the integration in the normalization factor (the total data likelihood). The technique of Markov (or Gibbs) sampling allows one to sidestep the complexities of the integration and to sample directly from the posterior likelihood distribution $P\left(\theta |D\right)$. This method follows from the observation that the normalization factor in 1 is a constant. The posterior is thus proportional to the product of the prior and the likelihood:

2 $$P\left(\theta\vert D\right)\propto P(\theta)\cdot P(D\vert\theta)$$

Sampling from $P(\theta)\cdot P(D\vert\theta)$ is therefore equivalent to sampling from the normalized posterior in 1).

The data likelihood $P(D|\theta )$ is customarily assumed to depend on the difference between data simulated by a model with parameters $\theta$ and observed data. In this study, it was defined as follows.

3 $$P\left(D|\theta \right)={e}^{-\frac{1}{N}\sum {\mathrm{ln}\left(\frac{{predicted}_{i}}{{observed}_{i}}\right)}^{2}}$$

Here, *N* is the total number of observed data points, ${observed}_{i}$ is the *i*th observed data point, and ${predicted}_{i}$ is the corresponding predicted data point.

An adaptive Markov chain Monte Carlo (MCMC) sampling method was employed to sample from the posterior likelihood, which was previously described by Vihola [27]. Since prior information on model parameters $P(\theta )$ was not available, the so-called uninformed prior (Jeffreys’ prior) was used [28], which initially places minimal constraints on the values the model parameters may take on. However, when considering the entire parameter space, sampling sufficient points to describe the posterior distribution proved to be prohibitively expensive, computationally. Therefore, upper and lower limits were set for each parameter. To identify reasonable limits, model parameters were manually optimized until the resulting model simulations visually resembled the observed biodistribution data to a reasonable degree. The upper and lower limits were then defined as a factor 30 higher and lower, respectively, than the manually optimized parameter values.

In addition to the upper and lower parameter limits, we also chose to only optimize a limited number of model parameters. These parameter values were chosen by inspecting their influence on the PBPK model outcomes based on the model equations. The following parameters, as well as the corresponding upper and lower limits used in the MCMC sampling method, are shown in Table 2.

**Table 2:** Model parameter chosen to be optimized, as well as their upper and lower limits used in the MCMC method

Parameter (unit)	Upper/lower limits (PACA-Cbz	Upper/lower limits (LipImage™ 815*)*	Description
χ_rich_ (-)	1.67 × 10^−2^–15	1.67 × 10^−1^–150	Permeability of richly perfused organs
P (-)	3.3 × 10^−2^–30	1.67 × 10^−3^–1.5	Tissue-blood partition coefficient
k_kidneyEl_ (h^−1^)	1–900	3.3 × 10^−2^–30	Renal elimination rate
k_ab0_ (h^−1^)	1.16 × 10^−2^–10.5	3.3 × 10^−2^–30	The maximum uptake rate of phagocytizing cells in organs except for the spleen
k_sab0_ (h^−1^)	1.67 × 10^−2^–15	3.3 × 10^−2^–30	The maximum uptake rate of phagocytizing cells in the spleen
k_de_ (h^−1^)	3.3 × 10^−5^–0.03	3.3 × 10^−5^–0.03	The release rate of phagocytizing cells

The remaining model parameters were adopted from Li et al. [26]. Although their study results are based on different NPs and exposure scenarios, the influence of the remaining model parameters on the model outcome is small. This assumption was verified using a local sensitivity analysis (results not shown). This local sensitivity analysis was performed by calculating the area under the curve (AUC) of the NBM amounts in the blood compartments (venous + arterial). In addition, the sensitivity analysis was performed for one parameter at a time; hence, the sensitivity of combined parameters was not assessed. Specifically, the sensitivity was calculated in the form of an elasticity coefficient S:

4 $$S=\frac{\Delta AUC/AUC}{\Delta p/p}$$

where $\Delta AUC$ is the change in the $AUC$ as a result of a change in the parameter value, $\Delta p$. Here, the applied change in the parameter values was always 10% of the original value,$p$, which means that $\frac{\Delta p}{p}=1.1$.

The PBPK model, the adaptive MCMC parameter sampling method, and the sensitivity analyses were implemented in R [29] using the packages mrgSolve [30] and adaptMCMC [31].

#### Model evaluation

A PBPK model was independently parametrized using the Bayesian parameter estimation approach for each of the five biodistribution data groups (i.e., the two dose groups of PACA-Cbz, and the three dose groups of LipImage™ 815). In order to quantitatively evaluate the quality of the PBPK model simulations, the average absolute fold error (AAFE) was calculated, which is defined as follows:

5 $$AAFE={10}^{\frac{1}{N}\sum \left|{\mathrm{log}}_{10}\left(\frac{predicted}{observed}\right)\right|}$$

where *N* denotes the total number of observations. Note that the AAFE was calculated separately for each parametrized model, and for each model compartment. An AAFE of 1 is achieved when the PBPK model simulation exactly matches the measured biodistribution data. The AAFE increases as the difference between the PBPK model prediction and the measured data increases.

### Results

**Fig. 2:**
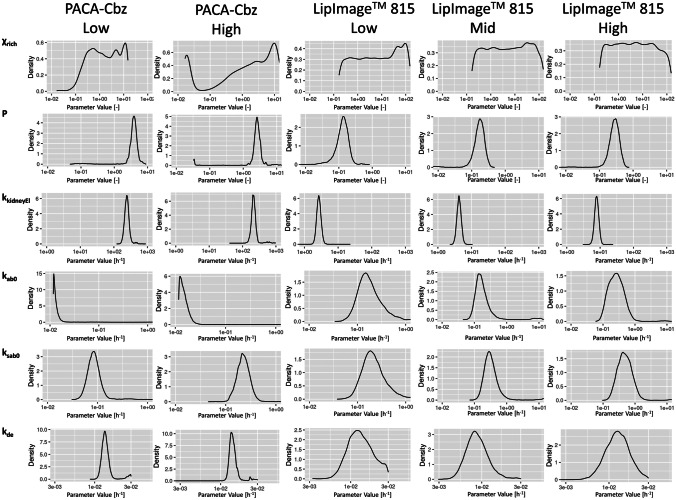
Probability density plots of the sampled posterior distributions of the six estimated parameters (i.e., χ_rich_, P, k_ab0_, k_sab0_, k_kidneyEl_, and k_de_) for the five in vivo biodistribution datasets

In the present study, a PBPK model was parametrized based on five in vivo biodistribution (sub)datasets concerning NBMs. Figure 2 shows the posterior distribution of each parameter estimated using the five biodistribution datasets. These posterior distributions are probability density plots $P\left(\theta |D\right)$ of the parameter values after considering the biodistribution data. This means that $P(\theta |D)d\theta$ gives the probability that a parameter has a value between $\theta$ and $\theta +d\theta$. As shown in Fig. 2, the posterior distribution of χ_rich_ is rather broad, covering at least two or more orders of magnitude in all five parametrizations. In addition, no clear maximum is observed in the posterior distributions of χ_rich_ for the LipImage™ 815 datasets. The posterior distributions of the remaining parameters were generally narrower (i.e., smaller than one order of magnitude) and always demonstrated a clear maximum.

In order to investigate whether PBPK parameters were correlated, pair-plots were made (supplemental material; Figures S1, S2, S3, S4, S5). Generally, no correlations were observed between the PBPK parameters. Only the macrophage uptake parameters, k_ab0_ and k_sab0_, seemed to exhibit a linear relationship when considering the parameter estimates based on the PACA-Cbz low-dose biodistribution data (Figure S1).

Table 3 shows the median value and the credible interval of the posterior distributions of all parameters and all five in vivo data subsets. The median value represents the most likely parameter value and will be referred to as the parameter estimate. The credible interval is the interval that contains 90% of the parameter values in the posterior distribution.

**Table 3:** Parameter estimates (median) and the corresponding credible interval (in parentheses) resulting from applying the Bayesian parameter estimation method using the five biodistribution datasets. *χ*_*rich*_, vascular permeability coefficient; *P*, partition coefficient; *k*_*kidneyEl*_, renal clearance rate; *k*_*ab0*_, uptake rate by macrophages (excluding those in the spleen); *k*_*sab0*_, uptake rate by macrophages in the spleen; *k*_*de*_, release rate by macrophages

Dataset	χ_rich_	P	k_kidneyEl_	k_ab0_	k_sabo_	k_de_
*PACA-Cbz Low*	**1.5** (1.6** × **10^−1^–1.3** × **10^1^)	**3.9** (2.4–5.4)	**2.4 × 10**^**2**^ (1.9** × **10^2^–3.2** × **10^2^)	**1.3 × 10**^**−2**^ (1.2** × **10^−2^–4.4** × **10^−2^)	**8.4 × 10**^**−2**^ (5.4** × **10^−2^–2.6** × **10^−1^)	**1.4 × 10**^**−2**^ (1.2** × **10^−2^–2.5** × **10^−2^)
*PACA-Cbz High*	**2.1** (1.7** × **10^−2^–1.3** × **10^2^)	**2.6** (1.7–4.0)	**2.1 × 10**^**2**^ (1.7** × **10^2^–2.8** × **10^2^)	**1.4 × 10**^**−2**^ (1.2** × **10^−2^–2.5** × **10^−2^)	**2.3 × 10**^**−1**^ (1.4** × **10^−1^–4.3** × **10^−1^)	**1.4 × 10**^**−2**^ (1.3** × **10^−2^–1.9** × **10^−2^)
*LipImage™ 815 Low*	**7.0** (2.4** × **10^−1^–1.2** × **10^2^)	**1.3** × **10**^**−1**^ (5.0** × **10^−2^–2.2** × **10^−1^)	**2.7** (2.1–3.4)	**1.7** × **10**^**−1**^ (8.3** × **10^−2^–8.0** × **10^−1^)	**2.1** × **10**^**−1**^ (1.0** × **10^−1^–8.7** × **10^−1^)	**1.3 × 10**^**−2**^ (7.6** × **10^−3^–2.6** × **10^−2^)
*LipImage™ 815 Mid*	**5.4** (2.4** × **10^−1^–11)	**1.7** × **10**^**−1**^ (8.4** × **10^−2^–2.8** × **10^−1^)	**4.3** (3.3–5.4)	**1.6 × 10**^**−1**^ (9.3** × **10^−2^–4.6** × **10^−1^)	**3.0 × 10**^**−1**^ (1.7** × **10^−1^–8.4** × **10^−1^)	**8.6 × 10**^**−3**^ (5.5** × **10^−3^–1.5** × **10^−2^)
*LipImage™ 815 High*	**4.5** (2.3** × **10^−1^–98)	**2.8 × 10**^**−1**^ (1.6** × **10^−1^–4.6** × **10^−1^)	**7.9** (6.2–10)	**2.6 × 10**^**−1**^ (1.1** × **10^−1^–6.4** × **10^−1^)	**4.7 × 10**^**−1**^ (2.2** × **10^−1^–1.1)	**1.3 × 10**^**−2**^ (7.0** × **10^−3^–2.2** × **10^−2^)

The results presented in Table 3 demonstrate that the estimated permeability of richly perfused organs, χ_rich_, and the estimated renal clearance, k_kidneyEl_, were approximately two orders of magnitude larger for PACA-Cbz than for LipImage™ 815. Similarly, the substance-blood partition coefficient, *P*, is approximately one order of magnitude larger for PACA-Cbz than for LipImage™ 815. The macrophage absorption rate parameters, k_ab0_, k_sab0_, and the macrophage release rate, k_de_, were comparable between PACA-Cbz and LipImage™ 815. Interestingly, *P*, k_sab0_, and k_kidneEl_ all increase with increasing LipImage™ 815 doses. Such a dose-dependent relation was only observed for the k_sab0_ when assessing PACA-Cbz.

**Fig. 3:**
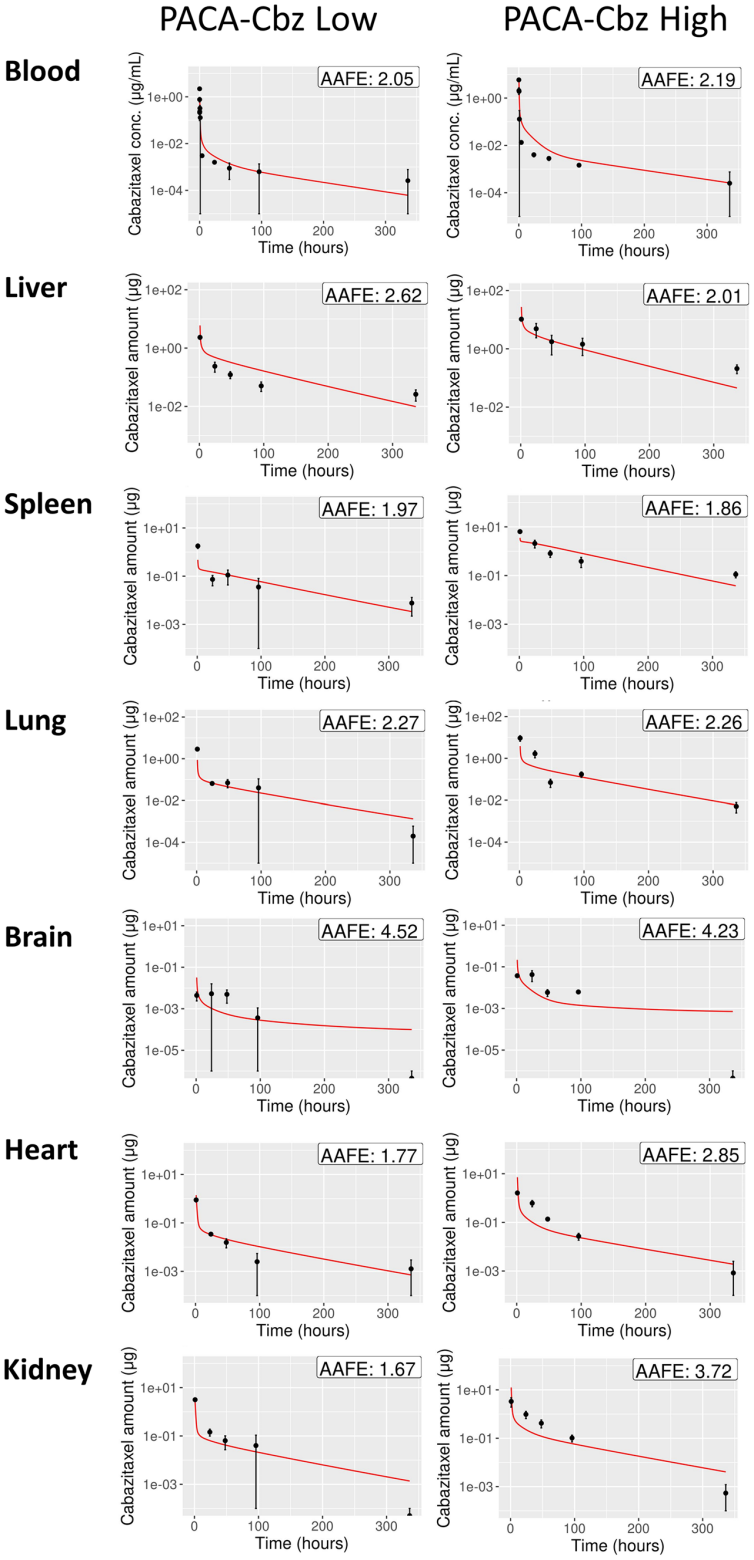
PBPK model simulation results (red) compared with measured cabazitaxel (black dots) in blood plasma, liver, spleen, lung, brain, heart, and kidney for two different doses. The difference between simulated and measured cabazitaxel levels, which is expressed with the AAFE, is shown in the right-upper corner of every plot. A larger AAFE corresponds with a larger difference, and an AAFE of 1 represents a perfect fit

The median parameter estimates shown in Table 3 were used to simulate cabazitaxel and IR780 levels. Figure 3 shows a comparison between the simulated cabazitaxel levels and those measured in vivo. The difference between the simulated cabazitaxel levels and the measured cabazitaxel levels was expressed with the AAFE, which is also shown in Fig. 3.

**Fig. 4:**
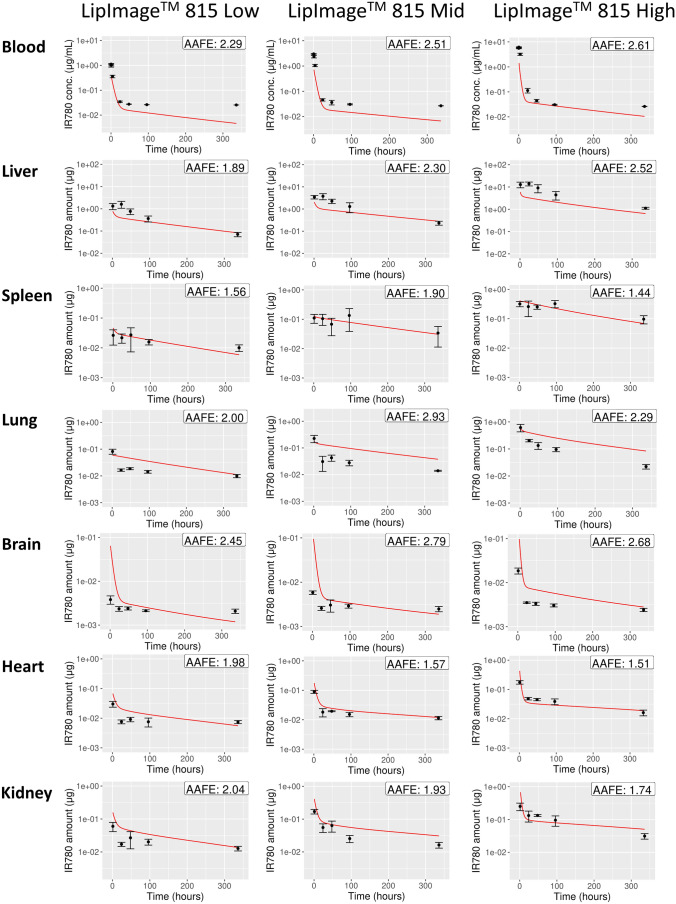
PBPK model simulation results (red) compared with measured IR780 (black) in blood plasma, liver, spleen, lung, brain, heart, and kidney for three different LipImage™ 815 doses. The difference between simulated and measured IR780 levels, which is expressed with the AAFE, is shown in the right-upper corner of every plot. A larger AAFE corresponds with a larger difference, and an AAFE of 1 represents a perfect fit

For each of the three LipImage™ 815 dose groups, simulated and corresponding measured IR780 levels are shown in Fig. 4. IR780 simulations are shown for the blood, liver, spleen, lung, brain, heart, and kidney. The difference between the simulated IR780 levels and the measured IR780 levels was expressed with the AAFE, which is also shown in Fig. 4. All AAFE calculated for IR780 were below 3, indicating an average discrepancy between model and data of less than a factor 3. In addition, the AAFE resulting from the spleen and the heart were always below 2, whereas the AAFE corresponding to the blood, lungs, and brain were higher than 2 for all three dose groups.

### Discussion

The use of NBMs has become a very promising approach to facilitate various medical applications including targeted drug delivery and medical imaging [32]. One of the key aspects for adequately using the NBMs is the optimization of the biodistribution of a substance by selecting the encapsulating NP [33]. The present study utilized a PBPK model to analyze the in vivo biodistribution of two NBMs after iv administration in rats. The NBMs considered in this work were PACA loaded with cabazitaxel particles and LipImage™ 815. Using a Bayesian parameter estimation technique, PBPK model parameters were estimated from in vivo biodistribution data. The Bayesian method is particularly suitable as it naturally accommodates the introduction of a priori information via a so-called prior. In principle, this method also allows the incorporation of initial model parameters measured in in vitro models. Ideally, an appropriate in vitro system may provide at least a correct order-of-magnitude estimate of a parameter’s value. This estimate may then be used to define a prior distribution, which can then be updated with, e.g., in vivo data. Limiting a search to a smaller region of the parameter space via an informed prior will significantly improve parameter estimation from distribution data.

The PBPK model parametrizations obtained in this study resulted in accurate simulations of PACA-Cbz and LipImage™ 815 concentrations. This indicates that the parameter estimation was successful. Interestingly, the parameter estimates resulting from the parametrization on the biodistribution data of PACA-Cbz were markedly different from parameter estimates resulting from the parametrization on the LipImage™ 815 biodistribution data (Table 3). More specifically, the estimated permeability of richly perfused organs, χ_rich_, and the estimated renal clearance, k_kidneyEl_, were approximately two orders of magnitude larger for PACA-Cbz than for LipImage™ 815, whereas the partition coefficient, P, was approximately one order of magnitude smaller for PACA-Cbz than for LipImage™ 815. Furthermore, the estimates of these three parameters based on PACA-Cbz fall outside the credible interval of the parameter estimated for LipImage™ 815. These findings may be (partially) explained by the different physicochemical properties of the two studied NBMs. Specifically, the zeta potential and the particle size substantially differed, which might have affected the biodistribution of the NBMs. This further emphasizes the need of tailoring the parametrization of PBPK models to the substance of interest. Conversely, the macrophage-related parameters k_ab0_, k_sab0_ (macrophage absorption rate), and k_de_ (macrophage release rate) seemed comparable among the considered NBMs. Whilst already known that macrophage uptake of nanoparticles is a key component of the biodistribution and biocompatibility of NBMs [34], the work presented here highlights the importance of clear determination of macrophage accumulation of NBMs, irrespective of the nanoparticle platform. Ongoing work within the REFINE consortium is aimed at defining, and translating methodologies to assess macrophage uptake of NBMs, that can aid in PBPK model development.

Information on PBPK model parameters as collected here will significantly aid the generalizability of PBPK modeling. If the database on specific model parameters is expanded, this could allow the grouping of NBMs according to kinetic parameters. Regression modeling or classification models could assist in predicting model parameters based on NBM properties, or at least assist in constructing prior distributions, thus greatly improving subsequent model parametrizations. Ideally, such a database could be supported by adequate in vitro measurements of kinetic parameters, further strengthening the development of informed, predictive priors for the parameter. An example of a PBPK model parameter that could be directly measured in in vitro systems is the permeability of the vascular epithelium (“χ_rich_”) [35]. In addition, NBM uptake by phagocytes (“k_ab0_”, “k_sab0_”) may be measured by incubating macrophages with fluorescently labeled NBMs and subsequently detecting fluorescence using a plate reader, flow cytometry, or confocal microscopy [36]. Future studies should be conducted to compare in vitro measurements of PBPK model parameters for the NBMs considered in this work, in order to evaluate the feasibility of the in vitro system for PBPK model parametrization.

Another important finding of this study was that certain parameters estimated using the LipImage™ 815 biodistribution data seemed to be dose-dependent. In particular, the partition coefficient P, the uptake rate of macrophages in the spleen, k_sab0_, and the renal clearance k_kidneyEl_ all increased with increasing dose (Table 3). In addition, minor to no overlap was found between the credible intervals of these parameter estimates between the different parametrizations. In contrast, the permeability χ_rich_, the uptake rate of macrophages outside the spleen k_ab0_, and the release rate from macrophages k_de_ did not demonstrate clear trends with increasing dose. This indicates that certain PBPK model parameters should be tailored to the desired LipImage™ 815 dose of the exposure scenario. The dose-dependency of certain model parameters might limit the extrapolation of LipImage™ 815 biodistribution data to different doses. Interestingly, for the PACA-Cbz biodistribution, this dose-dependency was only observed for the k_ab0_. Further research should be conducted to investigate whether the dose-dependency of PBPK model parameters is seen for other NBMs, and to identify other PBPK model parameters that might exhibit a dose dependency.

In the present study, a PBPK model was used to simulate the biodistribution of two NBMs. The biodistribution of the encapsulating NBMs is assumed to follow that of the substances loaded, since the biodistribution is mainly governed by the characteristics of the encapsulating NBM. However, this approach does not take into account the distribution of the free substances. As the encapsulating NBM degrades at a certain rate after administration, the loaded substance is released after which it may still migrate through the body, albeit with different kinetics than that of the NBM. In order to also consider the biodistribution of the free substance, a complementary model for the distribution and elimination of the free substance should be implemented and parametrized. Parametrization of this model would require measuring the encapsulated substance as well as the free substance. However, due to the technical challenges in making this distinction, there are currently no biodistribution datasets available that include this information. As a consequence, it remains challenging to incorporate the kinetics of the free substance into the PBPK model.

The present study shows that PBPK models can be well parametrized from biodistribution data for two NBMs, namely, PACA-Cbz and LipImage™ 815. In order to advance the applicability of PBPK modeling of the NBMs, data on specific PBPK model parameters should be expanded and preferably be compiled in a generally accessible database. Such a database would support the identification of groups of PBPK model parameters that are fairly constant across (a group of) NBMs, and of parameters that appear to be NBM-specific and require quantification on a case by case basis. Especially for the latter, the availability of adequate in vitro systems to estimate their values would greatly expand the generalizability of PBPK models. It should be noted, however, that a challenge in setting up such a database is that many different PBPK models have been developed, making the comparison between parameter estimates difficult. The database should therefore also include the model structure used to derive the parameter values. This model structure should be considered when searching for relations between estimated parameters and NBM properties.

Ideally, a PBPK model would be developed that predicts the biodistribution in humans, instead of animals such as rats. Although such human PBPK models have been developed for a few substances [37–41], validation of their predictivity is extremely challenging due to a lack of human biodistribution data involving NBMs. Instead of tackling this challenge at once, we aimed to first work towards a predictive model for rats. Such a model allows us to obtain a better understanding of the NBM properties in relation to the kinetic PBPK model parameters. The two most important steps to arrive at that stage are (1) the collection of more in vivo biodistribution data to expand the database and enable the identification of trends in kinetic properties and grouping of NBMs and (2) the development of robust and reliable in vitro methods that provide quantitative parameters that can be directly incorporated in a PBPK model. Although the second step is one of the objectives within the REFINE consortium, neither condition has yet been fulfilled. The present work contributes to the expansion of the database on kinetic parameters.

### Conclusion

This work presents the parametrization of a PBPK model on the basis of biodistribution data for two NBMs, PACA-Cbz and LipImage™ 815. Some of the identified PBPK model parameter values (i.e., the macrophage uptake and release parameters) were fairly constant over the different NBMs and dosing schedules. In contrast, parameters related to elimination, partitioning, and vascular permeability were clearly material-dependent. Overall, the parametrized PBPK model provided a good description of the distribution data, indicating the validity of the approach. However, the study also demonstrated dose-dependency in certain parameters when parametrizing the PBPK model using the LipImage™ 815 biodistribution data, which implies that the administered dose must be carefully considered when interpreting PBPK model simulations.

All in all, this study helps with expanding the dataset on PBPK model parameters for different NBMs. It should be noted that the parametrization was performed with the implicit assumption that the distribution is mainly driven by the distribution of the encapsulating NBMs, and that after degradation of the NBMs, subsequent re-distribution of the loaded substance does not occur to a significant extent. This introduces some uncertainty in the quantitative results. It is important to stress that future PBPK studies should ideally keep track of both NBMs and encapsulated drug. This would enable the development of generic, predictive PBPK models. This effort should be supported by the development of robust in vitro methods to quantify kinetic parameters. Some in vitro systems have already been developed for NBMs, but it is vital to understand variability in methodological approaches, to harmonize assessment of these key parameters. The results of this study furthermore show that the Bayesian parameter estimation method is a useful tool to parametrize a PBPK model. The Bayesian method is especially apt to combine information from different sources, be it different in vivo studies or in vitro data. Together with generic PBPK modeling, the Bayesian parametrization approach and in vitro kinetic models could greatly assist in the effort to replace, reduce, and refine (i.e., 3Rs principle) animal studies.

## Supplementary information

Below is the link to the electronic supplementary material.

Supplementary file1 (JPG 815 KB)

Supplementary file2 (JPG 730 KB)

Supplementary file3 (JPG 789 KB)

Supplementary file4 (JPG 873 KB)

Supplementary file5 (JPG 816 KB)
