# Supplementary figures and images for: Physiologically based pharmacokinetic modeling of intravenously administered nanoformulated substances
Source: Drug Deliv Transl Res. 2022 May 12;12(9):2132–44. doi: 10.1007/s13346-022-01159-w (PMC9360077; doi:10.1007/s13346-022-01159-w)

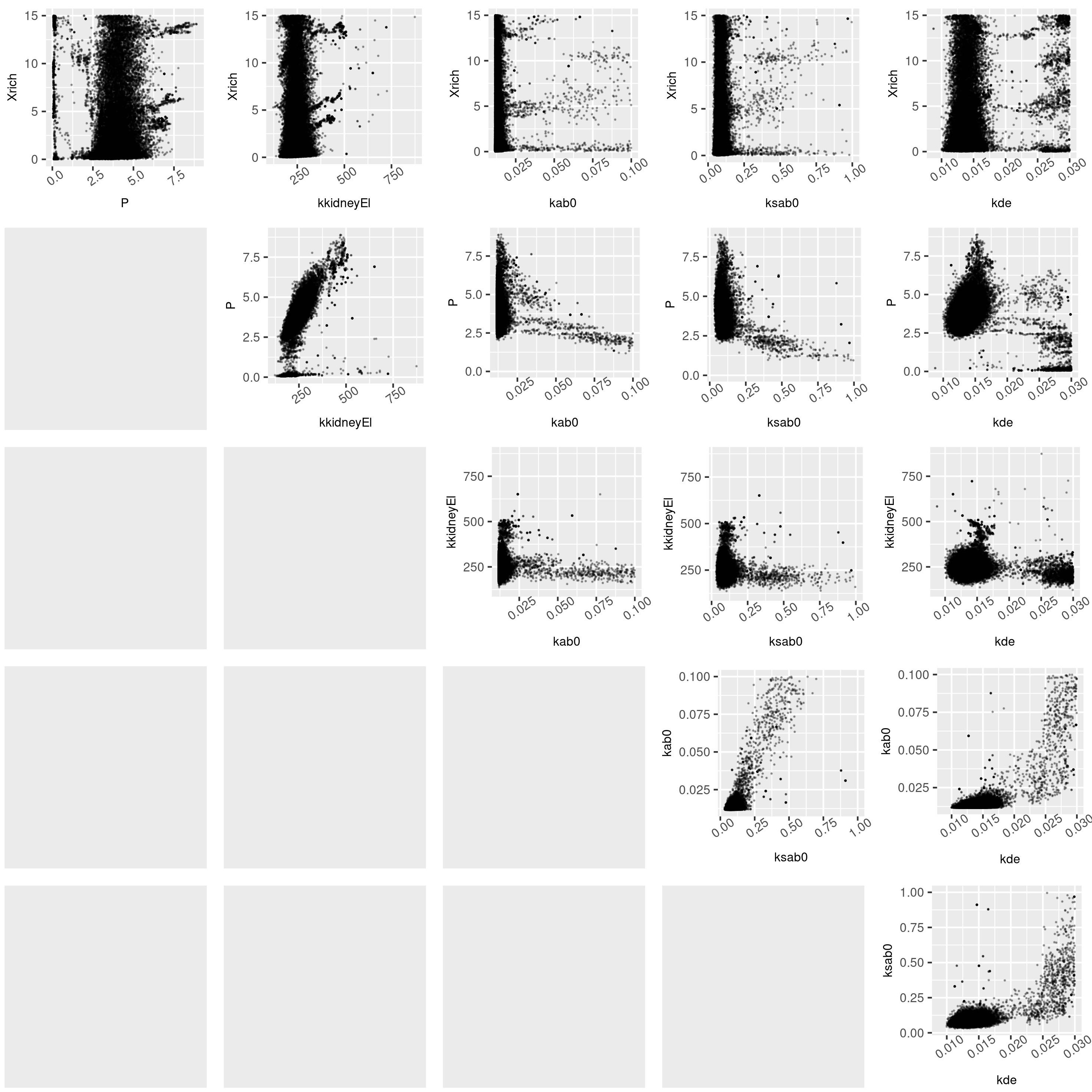

Supplement: Supplementary file 1 — Supplementary file1 (JPG 815 KB) [file 13346_2022_1159_MOESM1_ESM.jpg]

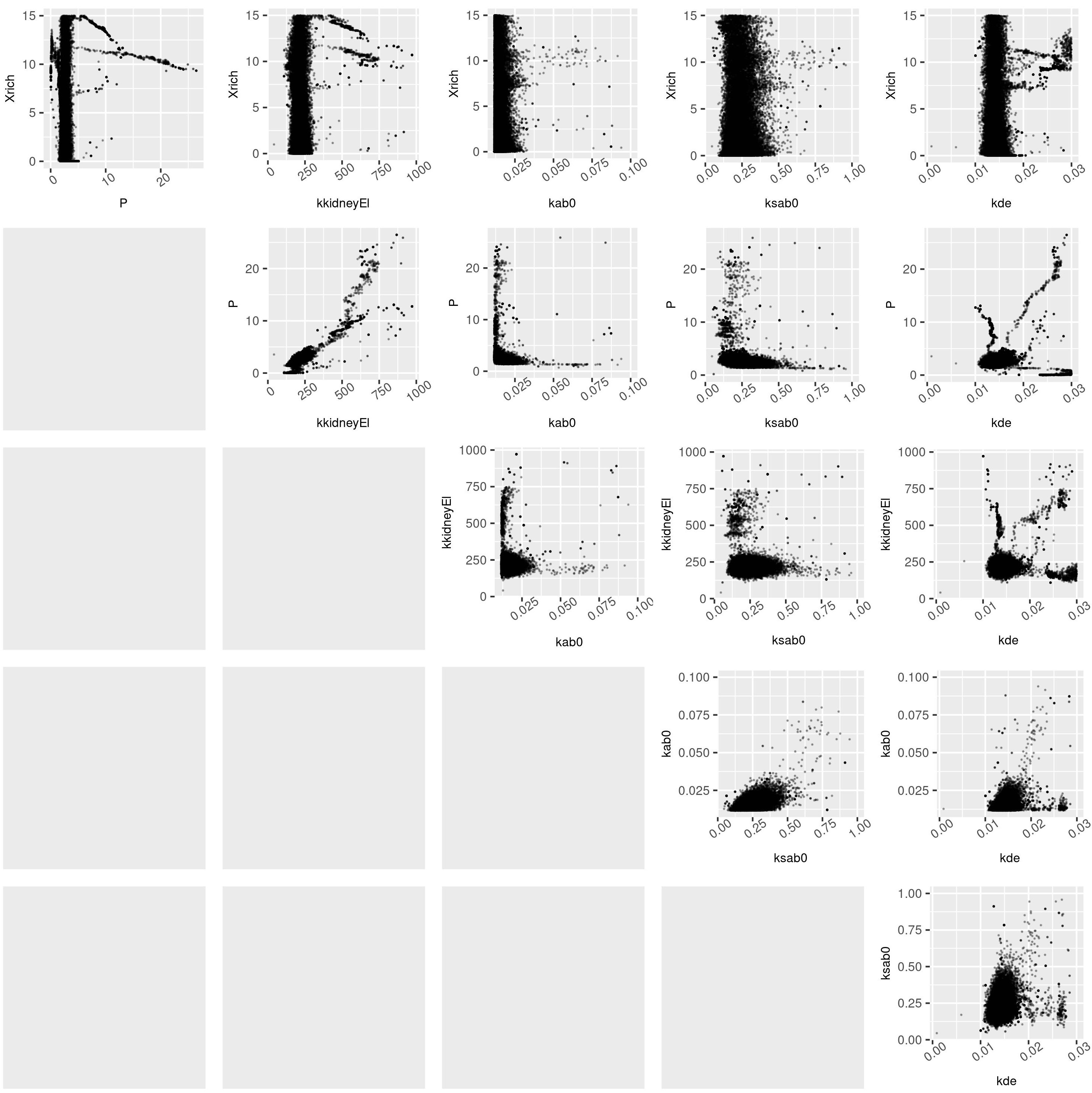

Supplement: Supplementary file 2 — Supplementary file2 (JPG 730 KB) [file 13346_2022_1159_MOESM2_ESM.jpg]

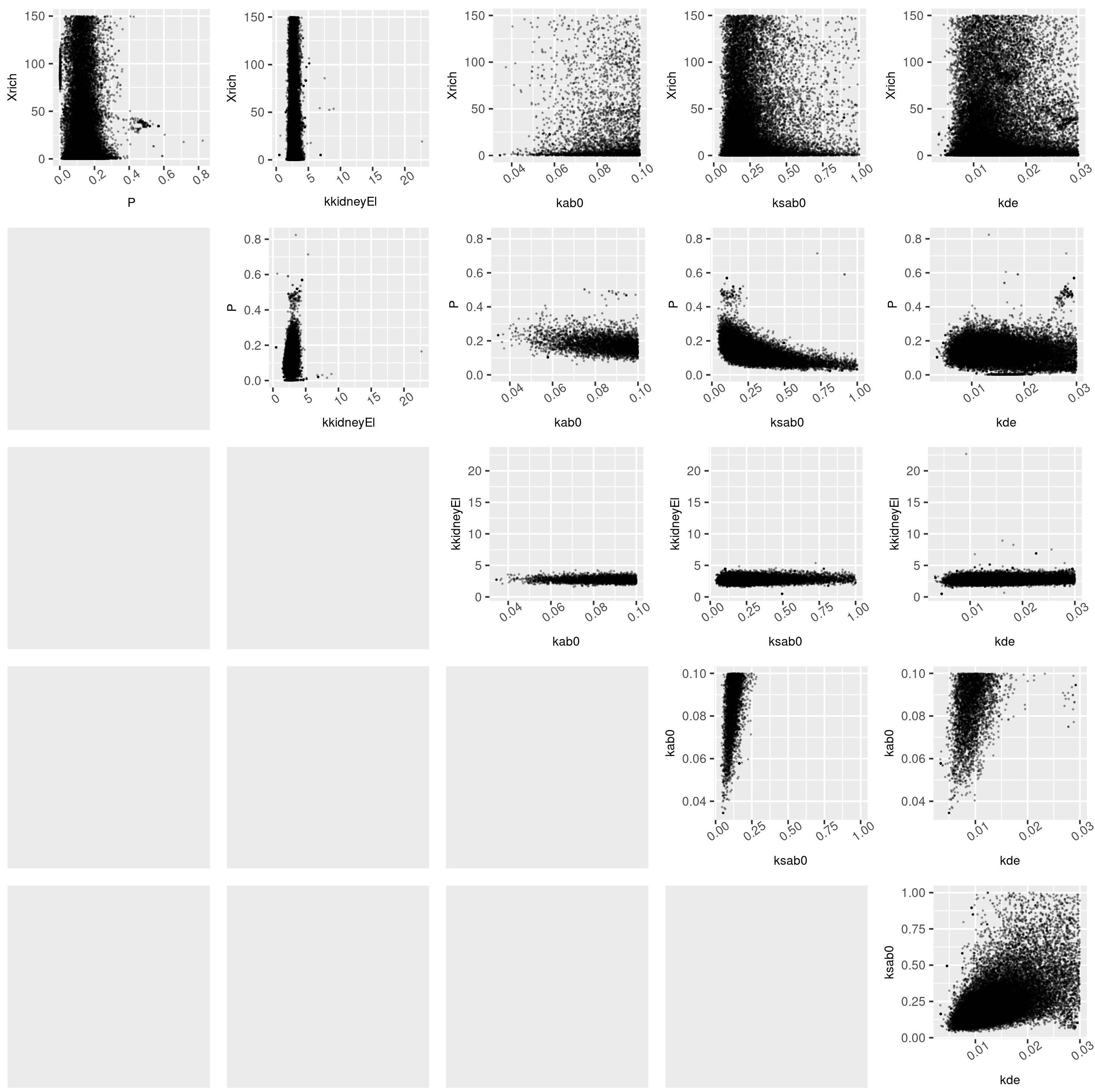

Supplement: Supplementary file 3 — Supplementary file3 (JPG 789 KB) [file 13346_2022_1159_MOESM3_ESM.jpg]

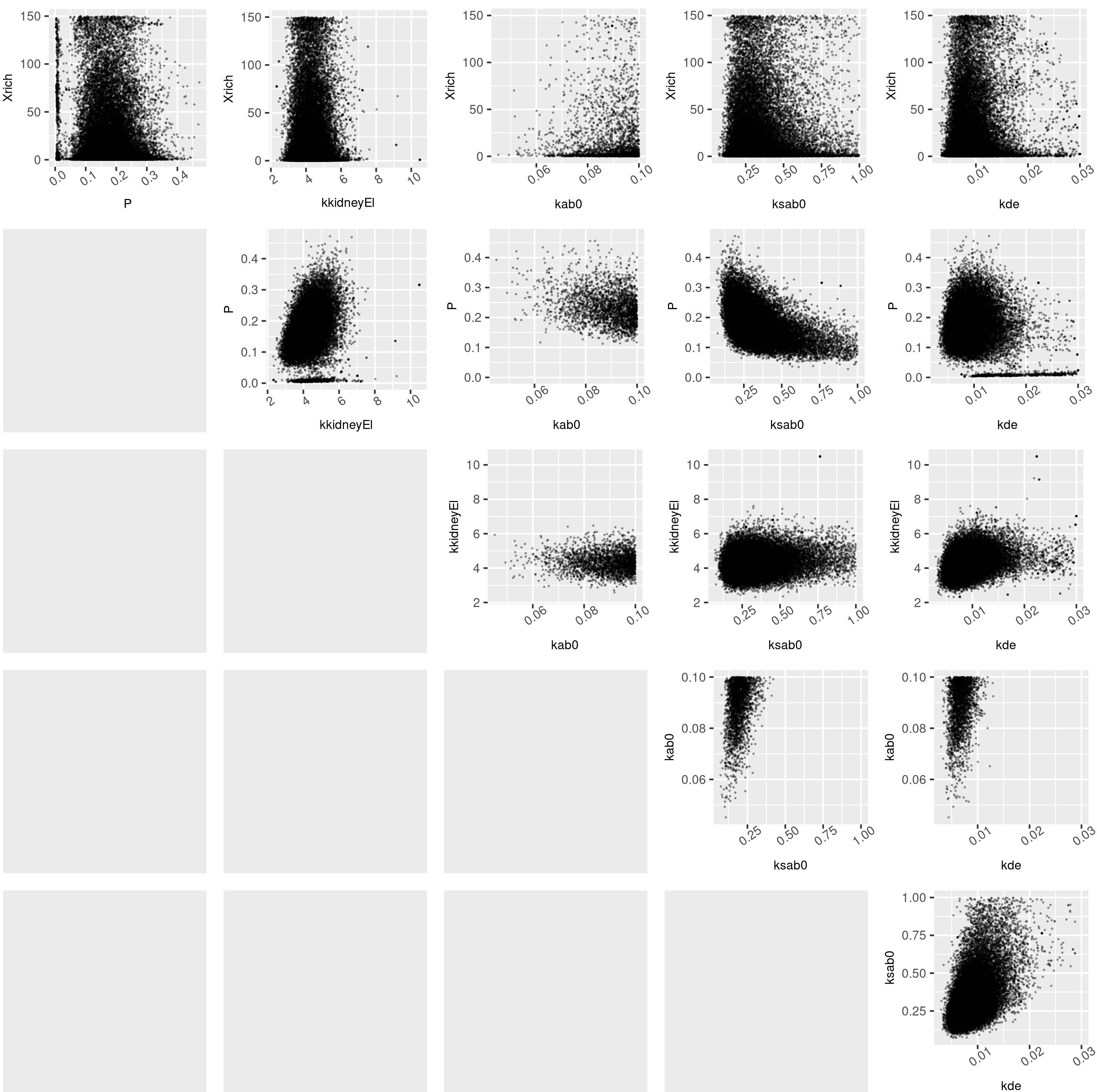

Supplement: Supplementary file 4 — Supplementary file4 (JPG 873 KB) [file 13346_2022_1159_MOESM4_ESM.jpg]

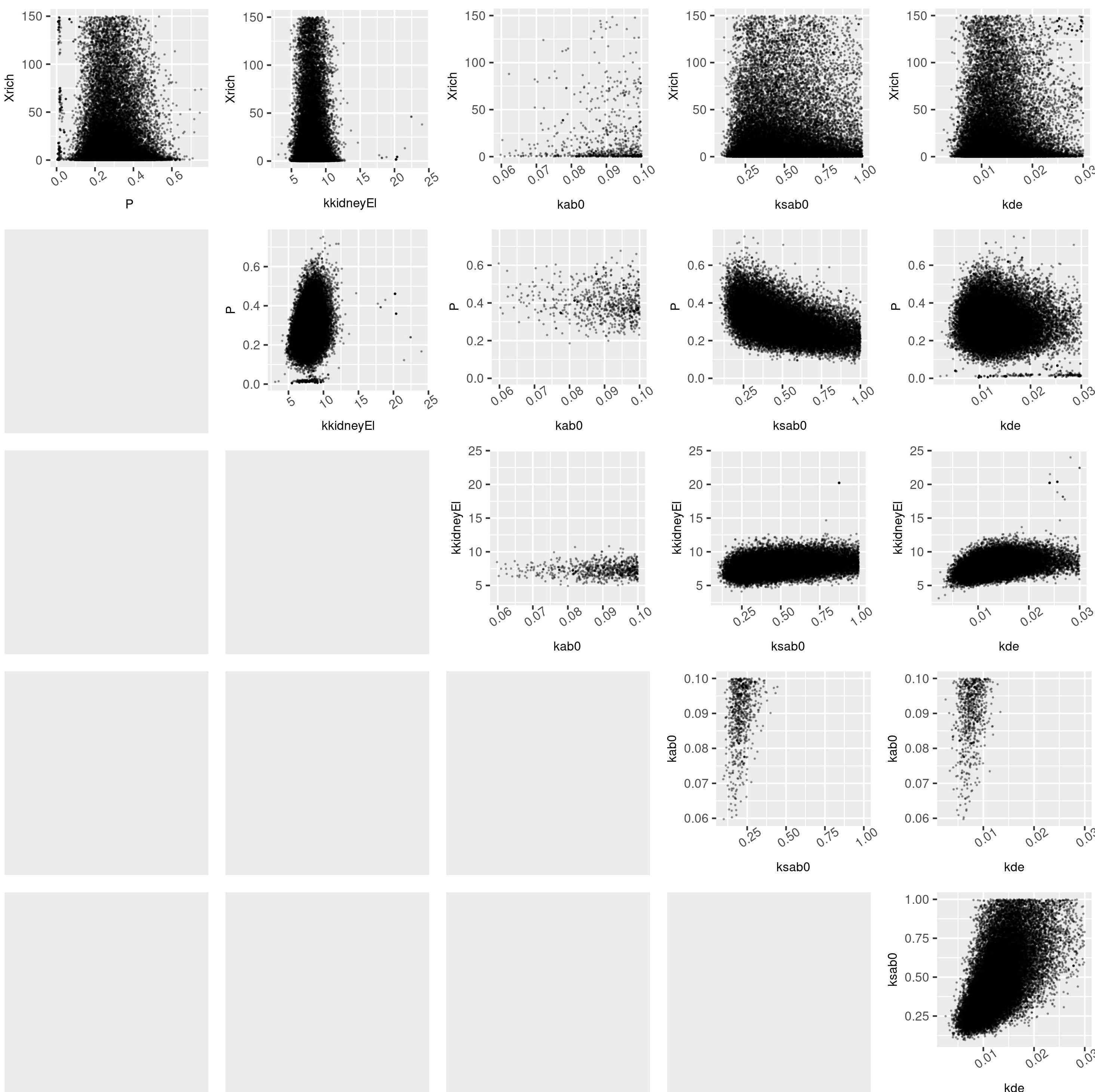

Supplement: Supplementary file 5 — Supplementary file5 (JPG 816 KB) [file 13346_2022_1159_MOESM5_ESM.jpg]
